# Sensors and Sensing Devices Utilizing Electrorheological Fluids and Magnetorheological Materials—A Review

**DOI:** 10.3390/s24092842

**Published:** 2024-04-29

**Authors:** Yu-Jin Park, Seung-Bok Choi

**Affiliations:** 1 Korea Initiative for Fostering University of Research & Innovation, Inha University, Incheon 21999, Republic of Korea; eugene5059@inha.ac.kr; 2 Department of Mechanical Engineering, The State University of New York, Korea (SUNY Korea), Incheon 21985, Republic of Korea; 3 Department of Mechanical Engineering, Industrial University of Ho Chi Minh City (IUH), Ho Chi Minh City 70000, Vietnam

**Keywords:** sensors, electrorheological fluid, magnetorheological materials, force sensor, tactile sensor, flux measurement sensor, strain sensor

## Abstract

This paper comprehensively reviews sensors and sensing devices developed or/and proposed so far utilizing two smart materials: electrorheological fluids (ERFs) and magnetorheological materials (MRMs) whose rheological characteristics such as stiffness and damping can be controlled by external stimuli; an electrical voltage for ERFs and a magnetic field for MRMs, respectively. In this review article, the MRMs are classified into magnetorheological fluids (MRF), magnetorheological elastomers (MRE) and magnetorheological plastomers (MRP). To easily understand the history of sensing research using these two smart materials, the order of this review article is organized in a chronological manner of ERF sensors, MRF sensors, MRE sensors and MRP sensors. Among many sensors fabricated from each smart material, one or two sensors or sensing devices are adopted to discuss the sensing configuration, working principle and specifications such as accuracy and sensitivity. Some sensors adopted in this article include force sensors, tactile devices, strain sensors, wearable bending sensors, magnetometers, display devices and flux measurement sensors. After briefly describing what has been reviewed in a conclusion, several challenging future works, which should be undertaken for the practical applications of sensors or/and sensing devices, are discussed in terms of response time and new technologies integrating with artificial intelligence neural networks in which several parameters affecting the sensor signals can be precisely and optimally tuned. It is sure that this review article is very helpful to potential readers who are interested in creative sensors using not only the proposed smart materials but also different types of smart materials such as shape memory alloys and active polymers.

## 1. Introduction

Recently, many smart materials have actively been studied as actuators and sensors for control applications such as automotive suspension, precision mechanisms and vibration control. Among many smart materials, both electrorheological fluid (ERF) activated by an electric voltage and magnetorheological materials (MRM) stimulated by a magnetic field intensity are very attractive candidates for various applications due to several inherent advantages including controllable apparent viscosity by the external stimuli, reversible phase characteristics, fast response time adaptable to most dynamic systems and easy design of feedback controllers associated with a semi-active actuating capability. In the early 1990s, the study of ERFs was more active than MRMs since the preparation of ERFs and the manufacturing of application systems was much simpler than those of MRMs. For example, the applications of ERFs only needs a positive and negative electrode to activate the fluid without any extra electric circuit. However, it has been recognized from several works that the field-dependent force (damping force) generated from ERFs due to the electrical field is too weak to apply to practical dynamic systems requiring a high damping force. Therefore, the dramatic change in the research trend from ERFs to MRMs arose in the early 2000s. It has been identified in several works that that MRMs have almost the same material characteristics as ERFs but produce a much higher field-dependent damping force (around 100–300 times) with relatively low power. Since then, numerous works on MRMs have been carried out to achieve enhanced field-dependent properties of the material itself as well as to increase the control performance of application systems. As a result, some commercialized products using MRMs are now available in several fields including automotive suspension dampers, automotive seat dampers and polishing machines for precise surface roughness. It is noted here that in this work MRMs can be classified as magnetorheological fluids (MRF), magnetorheological elastomers (MRE) and magnetorheological plastomers (MRP).

Review articles on ERFs published so far have considered several factors to enhance their field-dependent characteristics [1,2,3,4,5,6,7,8,9,10,11]. For example, several different particles such as alfa silica, alumina, polyurethane, mannitol, aluminum oleate, boron, carbon, colloidal silica, nylon powder and barium titanate have been mixed with various carrier fluids including silicone oil, transformer oil, dielectric oil, olive oil, castor oil, mineral oil, kerosene, grease and carbon tetrachloride. Several additives such as liquid crystals and surface coatings with a carbon nanotube have been also undertaken to increase the damping force and durability of ERFs. It is also noted that some fundamental theories regarding to the dynamic behaviors, the mechanism of chain-like formation depending on the magnetic field, the constitutive equations of the field-dependent rheology and the operating flow modes such as flow mode and shear mode have been studied in numerous works over two decades. Due to some benefits of ERFs such as fast response time and easy controllability of apparent viscosity, it has been applied to many systems of automotive shock absorbers, vibration control of flexible structures, medical devices (haptic master for surgical robot, knee rehabilitation orthotic, prosthesis), brake/clutch mechanisms, energy transportation and storage [12,13,14,15,16]. On the other hand, review articles on MRMs classified as MR fluids, MR elastomers and MR plastomers have been reported much more than those of ERFs, resulting in commercial products in the market, especially a vibration control area. Similar to ERFs, many investigations to enhance the MR effect have been performed by utilizing several particles (iron oxide, iron carbide, carbonyl iron, silicon steel, low carbon steel and nickel), various carrier liquids (silicone oil, polyalphaolefin, mineral oil, paraffin oil and aromatic alcohol), diverse additives (guar gum, antioxidant, metal oxide powders and viscosity modifier) and different surface coating materials (carbon nanotube, polyaniline, zirconia and polycarbonate) [17,18,19,20,21,22,23]. As for the application systems using MRMs, there are many review articles treating various systems or/and devices: MR dampers for automotive suspension system, MR dampers for civil engineering, large-sized MR mounts, control aspects of MRM application systems, energy harvesting MR dampers and vibration control of flexible structures [24,25,26,27,28,29,30,31,32,33,34,35,36]. However, most of the works on MRMs completed so far have been focused on the actuators or actuating mechanism instead of the sensors or sensing devices. This is because the desired force produced from MRMs can be easily achieved by applying an external magnetic field intensity. Therefore, the durability and predictability of control performances in application systems utilizing a MRM actuator are partially sufficient to meet a certain requirement as a commercial product. However, despite the numerous works on these smart materials, commercial products are strictly limited due to a couple severe problems: particle sedimentation and thermal effects during working operation [37,38]. This directly indicates that several challenging problems need to be resolved to use the smart materials considered in this review article as actuators or sensors.

It has been identified from the literature survey that even though some studies have been carried out on the possibility of ERFs and MRMs as part of a sensor’s fabrication, there is no review article focusing on sensors and sensing devices utilizing these smart materials. Consequently, the main technical contribution of this review article is to investigate and summarize recent works on the sensors and sensing devices which are available or realizable from ERFs and MRMs. This review is presented in a chronological manner and the sequence of the review is undertaken in the following order: ERF sensors, MRF sensors, MRE sensors and MRP sensors. In conclusion, after summarizing the review focusing on the inherent properties of each smart material, some challenging future works are briefly described to commercially realize the smart materials-based sensors which have high precision, easy calibration, high resolution and high accuracy in a real environment. In addition, a couple of modern technologies such as a neural network are suggested to optimally fabricate high performance sensors utilizing smart materials.

## 2. ERF Sensors

**Figure 1:**
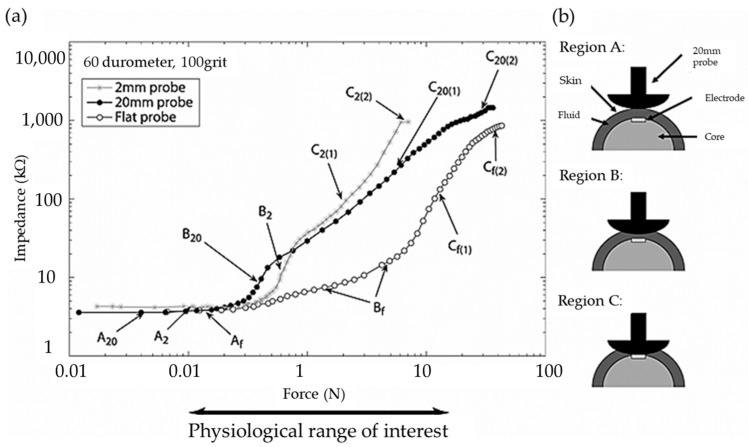
The tactile sensor array with three regions; (**a**) impedance (log scale) as a function of force (log scale) applied normally to the electrode surface. Textured silicone: 60 durometers, 100 grit size. (**b**) Graphic correlating curve shapes to probe indentation (2 mm probe shown). Reproduced with permission from [Nicholas Wettels], [Advanced Robotics]; published by [Taylor & Francis Group] (2008) [44].

**Figure 2:**
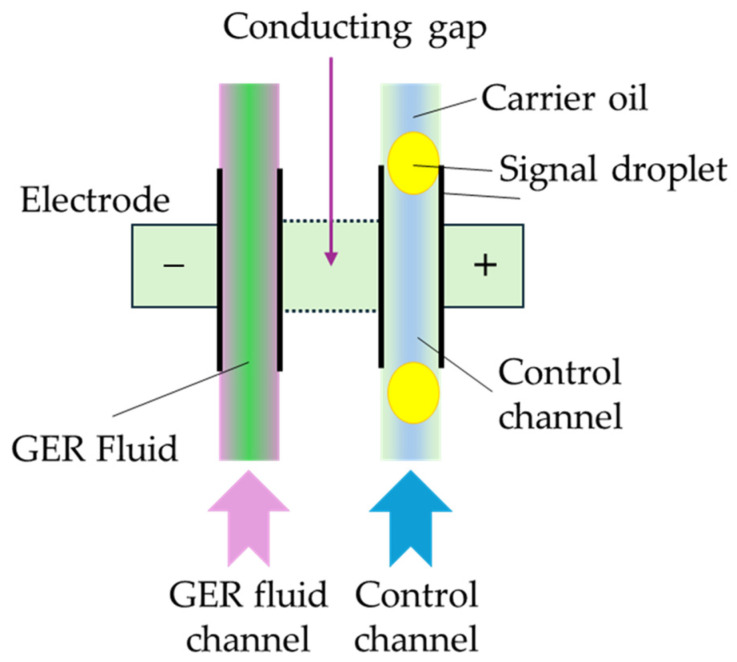
The fundamental logic-gate operation with a giant electro-rheological fluid [45].

As mentioned in the Introduction, an ERF is a kind of suspension consisting of fine dielectric particles and carrier viscous oils and hence it exhibits fast-changing rheological properties in the presence of an applied electric field. In other words, both the stiffness and damping properties are functions of the applied electrical field. Therefore, several sensors could be devised for the properties of ERFs. Kim et al. [39] devised a wave transmission sensor by embedding an ERF into the aluminum sandwich structures where a small-sized piezoelectric patch is used as a transmitter and receiver, respectively. It was found from this work that the magnitude and frequency of the transmitted signals and sensitivity of the device can be identified in the time or frequency domain. Han and Choi [40] devised a bi-directional clutch featuring a spherical ERF joint in which a torque is measured as a function of the field intensity. In this work, many data relating to the torque and field intensity needed to be collected as reference data and hence constant torque or sinusoidal torque depending on the field configuration was measured just like a fuzzy rule. Sagar et al. [41] measured a constant force in a shoe sole using the variable stiffness of an ERF. In this work, the sensing range of the force was achieved by changing the concentration of the copper particles. Then, the force was compared with the result measured from the universal testing machine. Zhang et al. [42] developed an integrated sensor using an ERF and conducting polymer (CP), where the ERF was used as an exciting actuator and the CP was used as a sensor. It was found that the exciting displacement was well measured up to 20 Hz, but when the vibration frequency was higher than 20 Hz the CP sensor could hardly detect the displacement. This kind of sensor mechanism has many potential applications, including ERF microvalves and a microchip with a CP sensor to achieve high control accuracy. Choi et al. [43] developed a speed sensor of a direct current (DC) motor by utilizing an ERF as a brake system. From the fuzzy table between the torque and motor speed as a function of the field intensity, the speed of the motor was measured for the torque response to the DC motor. In fact, this mechanism can be used as a torque sensor if the motor speed is measured by an encoder embedded in a servo DC motor. Wettels et al. [44] proposed a robust tactile sensor array which mimics the mechanical properties and also distributed touch receptors of the human fingertip using an ERF filled within an elastomeric skin. It has been found that the force ranged from 0.1 to 30 N by applying the electrical filed to the muti-electrodes. Figure 1 presents the schematic diagram for the proposed tactile sensor array in which three different regions representing different force levels are shown from the low force to the high force. Sheng and Wen [45] reviewed ERFs in terms of mechanisms, dynamics and microfluidics applications. They introduced a very interesting logic-gate operation device which can be used as a switch sensor in servo control systems. Figure 2 presents an operational principle of the logic-gate in which two parallel channels are separated by a conducting gap to pass the ERF and fluid signal. In this device, two electrodes are formed on the walls of two respective channels, while another conducting gap interconnects between the ERF channel and signal fluid channel, resulting in another electrode. Therefore, the signal indicating the fluid status can be tuned by an appropriate movement of the signal droplet.

**Figure 3:**
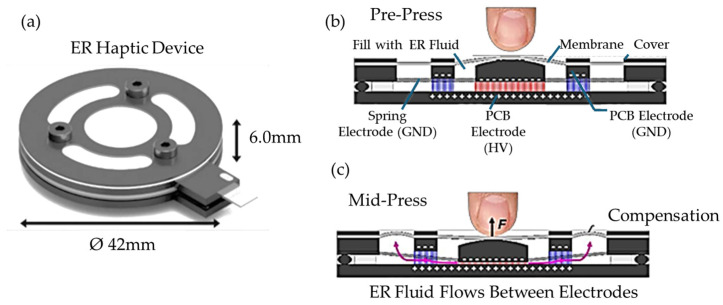
Schematic and working principle of a cylindrical ER device in which a user presses upon the membrane surface; (**a**) form factor of the ERF-based haptic device (cross-section view of the working principle of the cylindrical haptic actuator), (**b**) pre-press, (**c**) mid-press. Reproduced with permission from [Ping Sheng], [Annual review of fluid mechanics]; published by [Annual Review], (2011) [52].

Oh et al. [46] developed a haptic master which is applicable to minimally invasive surgery by robotic systems in which an ERF can sense the repulsive force occurring at the surgical point. By establishing the relationship between the generated force for four DOF haptic masters and the applied input current, the desired force is achieved from the signals from the load cell embedded in the haptic gripper, control current from the microprocessor and fuzzy logic based on the established table. Lee et al. [47] established a repulsive force feedback control system operated by a master-slave robot which is controlled by an ERF haptic master featuring a spherical joint mechanism. The force and rotational angle of the haptic master is measured by the load cell and encoder, respectively, and the robot arms are controlled by the servo motors. In order to measure the force of the skin cancer-like tissue (palpation) during the surgical operation, a phantom tissue is made and the force which is controlled from the haptic master by the electrical field is applied to the phantom to provide the desired force. This sensing system integrating an ERF-based haptic master whose force is controllable can be usefully applicable to robot-assisted minimally invasive surgery (RMIS). Liu et al. [48] proposed a tactile display to achieve a surface that can be felt by the human touch using the force responses from an ERF tactile array device. It was found from experimental tests that the sensed surface could be controlled by adjusting the input voltage and the touch sensitivity of the proposed tactile array was able to be applied as a surface touch sensor if the touching force normal to the display’s surface was well applied using a feedback control algorithm. Yoon et al. [41] have shown that ERFs are able to respond to light and electric fields by mixing various photosensitive molecules. Therefore, the newly formulated ERF can be treated as a new type of photocell which can be applicable to the basic elements of many sensors based on electric circuits producing the out current, voltage and resistance. However, it is possible to be made when the effect of the photo additive only can be identified from the field-dependent rheological properties. Chou et al. [49] proposed an electrorheological display (ED) to prevent a plague which transmits quickly to a variety of wildlife rodents. In order to achieve this goal, they made EDs consisting of two indium tin oxide glasses with spacers to contain core-shell structured polystyrene microspheres (SMs) by emulsion polymerization to absorb the magnetic nanoparticles (FNs) to encapsulate dispersed SM@FN. Then, the transmittances of ED-loading SMs@FNs can be identified as a function of the frequency at a certain voltage-on and hence the transmission of the plague may be protected. Mazursky et al. [50] embedded a sensing mechanism using an ERF in which tactile sensations at a small scale are conveyed from the field-dependent resistive force of the haptic feedback system. They made a deflection sensor and verified its effectiveness where the sensor design was created on the basis of the stress-sensitive resistive film in bending to the ERF haptic actuator. Jekal et al. [51] introduced ERFs which have different colors with specific properties and applications. They made a series of TiO_2_-coated synthetic mica materials colored white, yellow, red, violet, blue and green using a facile sol-gel method that can be identified by the naked eye. Therefore, users can easily select the proper ERF by observing the color in which many data such as the field-dependent yield stress, sedimentation stability, particle sizes, carrier liquid, conductivity and dispersion stability are provided in a standard manner. It is noted that the colorful ERF is not a sensor but plays a role in the visual selection to achieve the desired field-dependent characteristics. Mazursky et al. [52] proposed a miniature haptic module based on an ERF to convey both combined stiffness and vibrotactile sensations at a small scale. Figure 3 shows the schematic and working principle of a cylindrical ER device in which a user presses upon the membrane surface; the grounded spring electrode is displaced vertically by squeezing the ERF between the grounded spring and high voltage electrodes. The fluid flows radially through parallel electrodes by the pressure gradient from the press and hence the membranes between the slots are deformed elastically. Thus, by applying the voltage to the electrodes, a microstructural deformation occurs due to the force, which is felt by the user’s finger. It is noted that the force is a function of the pressure and the applied voltage and hence an appropriate dynamic range of simultaneous kinesthetic and tactile sensation can be devised for several applications, including the surgical robot finger.

Salunkhe et al. [53] presented the general properties and applications of ERFs, showing the recent status of the science and technology. They asserted that ERFs have potential applications as sensors, rollable screens and keypads sticks. The ERF sensors are active sensors which are generally used for the self-monitoring and control of vibrations in several flexible structures such as building structure design. Using the same concept as above, the ERF can be used as a detector of seismography or working performance of bridge health. Pavlikova et al. [22] developed a new type of ERF using a nano-silica grafting approach to address the drawbacks of conventional ERFs consisting of carbonyl iron particles which possess high electrical conductivity and hence the limitation of the field-dependent rheological characteristics. The carbonaceous particles treated by thermal carbonization in an inert atmosphere were coated with mesoporous nano-silica to obtain the semi-conducting particles. It was identified in this work that the conventional ERF caused a short circuit of the measuring device at the electric field of intensity 1 kV/mm, while the proposed ERF was successfully measured up to 3 kV/mm, giving less thermal conductivity, higher sedimentation stability and higher yield stress than a conventional ERF. Therefore, from the enhanced performances, the coating material amount, coating thickness and dielectric property of the coating material could be identified by the fuzzy table in which the relation between the coating specifications and rheological performances are summarized as a function of the electrical field intensity. Musialek et al. [54] estimated design parameters of a viscous clutch system using a transfer function which contains the principal dynamic parameters of a hydraulic viscous clutch operated and controlled by an ERF. The mathematical transfer function of the system was formulated as the first-order system with the time delay and the prototype of the system was tested. It has been found that the simulated operation with the estimated parameters is the same as the one measured with high accuracy under external disturbances. Therefore, the principal design parameters such as the orifice diameter of ERF flow could be identified from the proposed approach. Spotowski et al. [55] formulated a sensing mechanism using a strain gauge sensor and viscous brake filled with an ERF to measure the impact of the rotational speed of the input shaft. In other words, this device measures the constant pressing force exerted on the ERF brake connected to the shaft of the electric motor and thus different values of the pressing force can be obtained by changing the angular velocity of the motor as well as applying different voltages to the electrodes. Therefore, the final target of this work is to make a device to exert a constant pressing force (DECPF) to measure the force which is dependent on the angular velocity of ERF brake and the level of voltage applied to the electrodes. This kind of sensing mechanism is applicable to optimally control the braking force of many types of brakes and clutches. Recently, Liang et al. [56] reviewed the ERF technology in terms of the fabrication of the ERF itself, material characterization, constitutive models, coil structures, energy-related issue, various applications and the introduction of an electrorheological elastomer (ERE) with sensor applications such as a force sensor and speed of table tennis.

## 3. MRF Sensor

**Figure 4:**
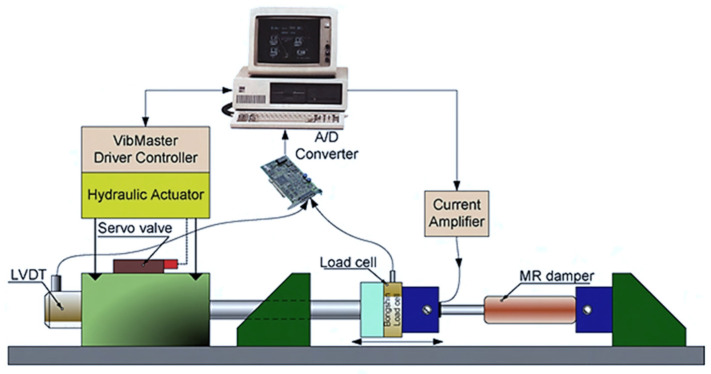
Diagram of a self-sensing behavior with fuzzy mapping for identification of MR dampers [59]. Reproduced with permission from [Dinh Quang Truong], [Sensors and Actuators A: physical]; published by [Elsevier], (2010).

**Figure 5:**
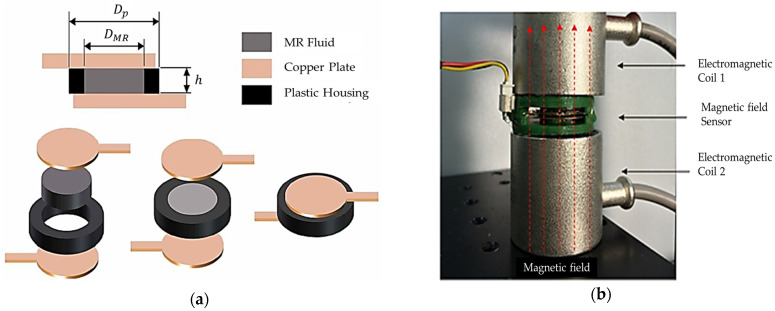
Configuration of the flux measurement sensor; (**a**) configuration of the MRF-VR system, (**b**) photograph of the measurement system in magnetic field calibration unit [60,61]. Reproduced with permission from [Suresh Kaluvan], [International Journal of Mechanical Systems Engineering]; published by [Graphy Publications], (2015), (**b**) Reproduced with permission from [Suresh Kaluvan], [Sensors and Actuators A: Physical]; published by [Elsevier], (2016).

**Figure 6:**
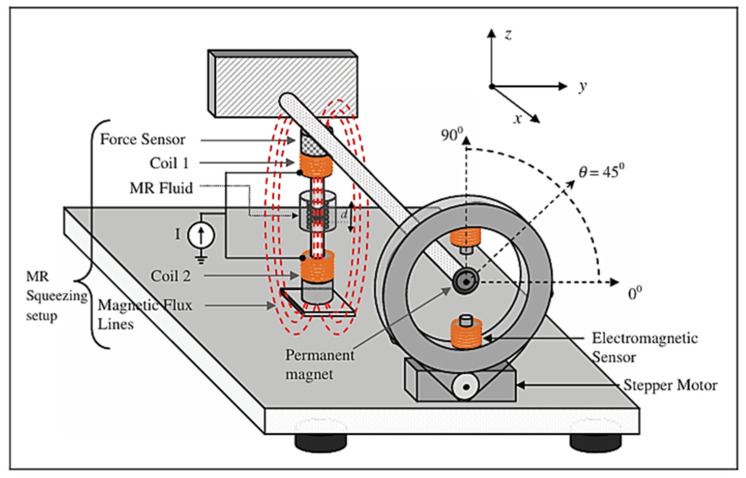
Schematic representation of the dynamic motion measurement system using a MRF [62].

The field-dependent rheological properties of MRFs such as the yield stress are much higher than those of ERFs and more stable since they are magnetic sensitive instead of high voltage. Therefore, MRFs as actuators or sensors have a wider range than ERFs. Flores and Liu [57] demonstrated the possibility of using MRFs as in vitro cancer therapy devices by mechanically blocking the blood vessels to a tumor. In this work, a simple blood network consisting of four branches of blood vessels, where one of branches is used as a container of the blood vessels with the cavity and either two or four vessels are subjected to a magnetic field, was created. Then, the weight of the leaking blood downstream from the magnets was measured to judge the field intensity for the cancer therapy. Jung et al. [58] investigated the sensing ability of an electromagnetic induction (EMI) system connected to MRF dampers for vibration control. Thus, according to Faraday’s law of electromagnetic induction, the emf signal, produced from the EMI, is proportional to the velocity of motion. Therefore, the induced emf voltage signal is converted to the velocity signal generated by the mechanical shaking table. Troung and Ahn [59] proposed a black-box model (BBM) for identification of MR dampers using self-sensing behaviors in which a fuzzy mapping system was used to estimate the damper’s characteristics. In order to improve the accuracy of the suggested model, the back propagation learning rules based on the gradient descent method were utilized to train the fuzzy parameters to minimize the modeling error function. Then, the proposed BBM with the optimized parameters could be used as a virtual sensor to measure the damping force of a vibration control system equipped with a MR damper. Figure 4 shows the schematic configuration and working principle of the proposed sensing mechanism. It is seen from the figure that a linear variable differential transformer (LVDT) is used to measure the displacement of the piston rod of the MR damper, while a load cell is attached to measure the field-dependent damping force. Then, the field-dependent damping force can be measured from the back signals measured by the LVDT and load cells and processed in the data acquisition system as input and output signals. Kaluvan et al. [60] developed a new measurement method for the field-dependent yield stress of MRFs using the resonance concept of the beam. The resonance frequency of the cantilevered beam was changed due to the magnetic field intensity and hence the change of the yield stress could be measured by analyzing the shifted in resonance frequency. Kaluvan et al. [61] surveyed the sensors and sensing networks made from MRFs. As a first example, the resonant sensor was introduced in which the output frequency was changed or tuned as a function of the physical parameters of the MRF, such as viscosity. The proposed method is accomplished by utilizing the resonant behavior of the cantilever structure associated with the piezoelectric excitation and detection. Secondly, a new type of current sensor using a MRF was discussed in the oscillatory shear motion. The change in resonant frequency of the cantilever beam due to the shear mode rheological effect of the MRF for the corresponding input current was measured, followed by formulating the relationship between the resonant frequency and input current. Moreover, the magnetic flux sensor could be devised using MRF. Figure 5 presents the configuration of the flux measurement sensor. As seen from Figure 5a, the measurement device produces the different signals depending on the thickness of the MRF, which directly indicates the change in the variable resistor (VR) and hence variable rheological properties depending on the current level (or magnetic field intensity). The mechanism of the MRF-VR is produced on the basis of the interaction between the ferromagnetic iron particles of the MRF which causes the electrical resistance, which is field-dependent. Two copper plates are attached to the top and bottom of the plastic housing fully filled with the MRF. The variables of H and $D_{p}$ are the height and diameter of the plastic housing, respectively, and DMR is the diameter of the contact area between the electrodes and MRF. In fact, it has been identified from an experimental test, shown in Figure 5b, that the initial resistance (maximum) for the two MRF-VRs is 104 and 17.58 MG (MΩ), respectively, and the final values (minimum) are measured as 4.46 and 2.09 MΩ. When the contact area is larger, more electrons easily pass the MRF through the higher number of iron particle bridges generated between the two electrodes. Kaluvan et al. [62] developed a new measurement method to achieve the dynamic signals of MRF subjected to a squeeze operation mode as shown in Figure 6. The disc-type permanent magnet is attached to the free end of the cantilever rod and an electromagnetic actuator is placed near to the permanent magnet. The MRF is placed between the gap to form the squeeze effect, and then the horizontal direction of the vibration of the cantilever rod produces the shear mode operation in the MRF squeezing setup. Similarly, both the vertical and intermediate directions of the rod vibration produce the squeeze and coupled mode operations. Therefore, the field-dependent dynamic behavior of the MRF is measured for several directions of the actuation angle. More specifically, the dynamic force and vibration amplitude reduction at different operation modes of the MRF can be measured from the measurement methodology.

**Figure 7:**
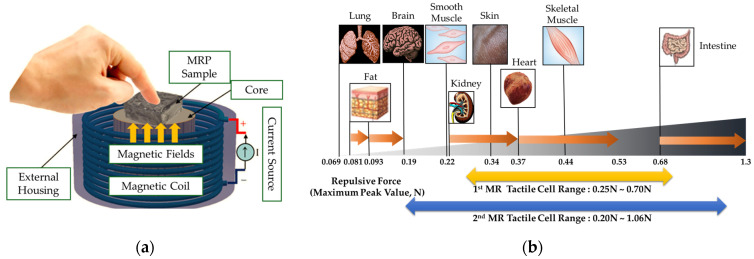
Tactile force sensor and sensing spectrum; (**a**) schematic of MRP sample, (**b**) force spectrum of MRP tactile device [66,67].

**Figure 8:**
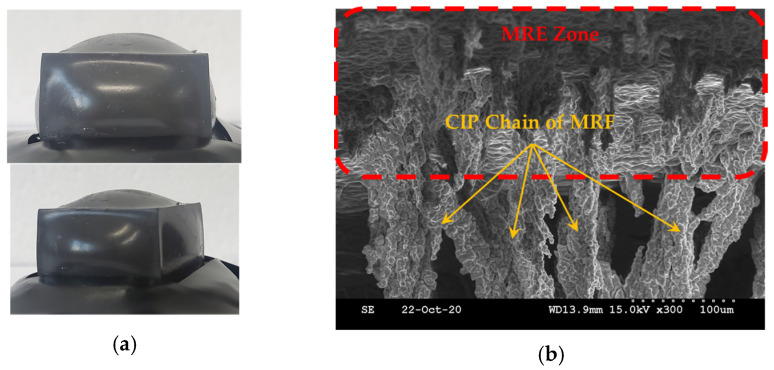
Dynamic sensor mimicking human organs; (**a**) fabricated sensor sample, (**b**) the microstructure inside of the sensor—the shape of the MRF chains extending from the MRE [69].

**Figure 9:**
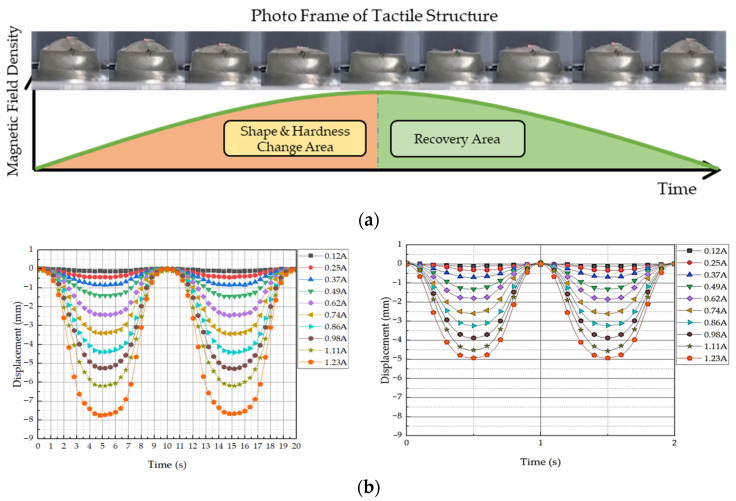
Dynamic motion tactile sensing device; (**a**) schematic of dynamic shape by the input magnetic field, (**b**) the field-dependent dynamic motion results in 0.5 Hz of excitation [69].

Kim et al. [63] devised a new tactile device using a MRF sponge cell to realize the viscoelastic sensation of a human’s real organs, such as the stomach, in which the surgeons can directly contact the sponge cell during the robot surgery. The effectiveness of the proposed device has been demonstrated by measuring the relation time, bending moment and repulsive forces at various input currents and comparing them with the existing values corresponding to each human organ. Pepe et al. [64] presented an electronically controlled suspension system MR damper installed on a real car which relies on a sensor network capable of acquiring a large real-time dataset collecting the car vibration and trim. The information on the car is elaborated by an electronical controller based on a new controller, variational feedback control (VFC), which drives a set of semi-active MR dampers. In the real time realization, a number of key performance indexes such as high ride comfort and limited vertical acceleration are considered to emphasize how the VFC is able to sneak into problematic regions of the performance planes because they are characterized by different and antithetic features of the suspension system. It is noted here that this article does not present the MFR sensor directly. But, it shows the sensing network of the feedback control system associated with the MR damper. Li et al. [65] proposed a new displacement sensor by embedding a soft MRF film with ferromagnetic particles which can induce scattering on the evanescent field of a planar waveguide at a proximity distance. This distance can be controlled precisely by the magnetic intensity, showing a maximum sensitivity of ~2.62 dB/T. Park et al. [66] proposed a controllable tactile device capable of realizing repulsive forces from human tissues using a MRF with porous polyurethane (PPE). It is called a MRP tactile sensor. In the fabrication process of the samples, the MRF was immersed into PPE and tested under the squeeze mode. Figure 7a presents a simple schematic configuration of the proposed tactile device in which the hand of the tester is located in the same axis as the direction of the magnetic field. Thus, the repulsive force measured from the tactile force can be controlled by the magnetic field intensity. Figure 7b represents the relationship between the maximum repulsive (or peak) values achieved from the proposed MRP and the stiffness of the human tissues. It is seen that the sample of MRP2 which has a 100 ppi PPE has a wider range covering more human tissues than MRP1 which has a 25 ppi PPE. It is noted that a tactile device covering all human tissues could be made by adjusting the particle concentration and the magnetic field intensity. Park and Choi [67] presented a new type of tactile transfer cell using a MRF which can be effectively applied to robot-assisted minimally invasive surgery. In fact, the proposed tactile device consists of two smart materials: a MRF and magnetorheological elastomer (MRE), whose viscoelastic properties are controllable by an external magnetic field. The relationship between the field-dependent repulsive force and compressive deformation depth is formulated to achieve the field-dependent Young’s modulus utilizing the finite element method. It has been confirmed that the proposed tactile transfer cell can mimic the repulsive force (hardness) of several human organs. Park et al. [68] presented a dynamic tactile device utilizing the spherical shape of a MR structure whose dynamic motions of magnitude and frequency can be controlled by the magnetic field intensity. After manufacturing a prototype, a sinusoidal magnetic field which has different exciting frequencies and magnitudes was applied to the sample, and then the dynamic motion of the contraction and relaxation depending on the exciting magnetic field was observed. As one of test results, when a 10% deformation occurred, the instantaneous force was generated from 2.8 N to 8.8 N and the force when relaxed was measured from 1.2 N to 3.5 N. It has been also shown that the repulsive force within this range can be implemented using the acceptable input current. Such a special tactile sensing structure proposed in this work can be used as a sensor to measure the field-dependent viscoelastic properties of human tissues such as the stomach, liver and body. In addition, it is usefully applied to robot surgery because it can mimic the dynamic motions of various human organs under various surgical conditions. Park et al. [69] presented a new sensor mimicking the dynamic contraction and relaxation motions of human tissues utilizing both MRFs and MREs. As for the MRF, MRF-122EG produced by Lord Corporation (Parker Load, Cary, NC, USA [70]) was used and a MRE was fabricated by mixing the CIPs with silicone rubber based on a 3D printed mold and undergoing a curing process for 4 h. Figure 8a presents a sample of the fabricated sensor and Figure 8b shows the inside microstructure in the presence of the magnetic field. It is expected from the figure that the CIP chains within the MRF extend from the surface zone of the MRE as a function of the magnetic field intensity. Therefore, the proposed sensor can change the dynamic shape by the input magnetic field. In other words, the magnitude and frequency of the sensor can be controlled by applying the corresponding input magnetic field. Figure 9a shows one of dynamic motion results under the influence of the magnetic field during a 1/2 frequency cycle. It is clearly observed from the figure that as the magnetic intensity increases, the maximum shape change (maximum contraction) occurs due to the increment of the downward and sideways pulling force. If the strength of the magnetic field is off, the original shape is restored as it enters the recovery zone. Therefore, the time-dependent and field-dependent stress can be considered as the relaxation of the viscoelastic property. This dynamic motion can be represented by the displacement change as a function of the exciting frequency as shown in Figure 9b. It is observed that as the input current increases, the displacement is increased at the interval and the displacement (or deformation) of the proposed sensor is decreased as the input frequency increases. Therefore, the dynamic motions of various human organs which have different amplitudes and frequencies can be realized using the proposed sensing device.

## 4. MRE and MRP Sensors

**Figure 10:**
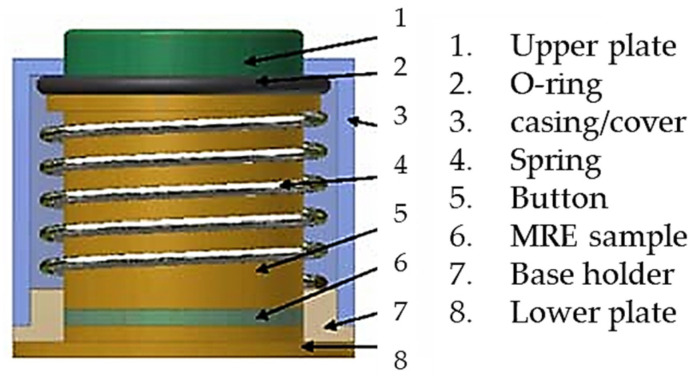
Schematic design of the MRE force sensor [74]. Reproduced with permission from [Weihua Li], [International Conterence on Advanced Intelligent Mechatronics]; published by [IEEE], (2009).

**Figure 11:**
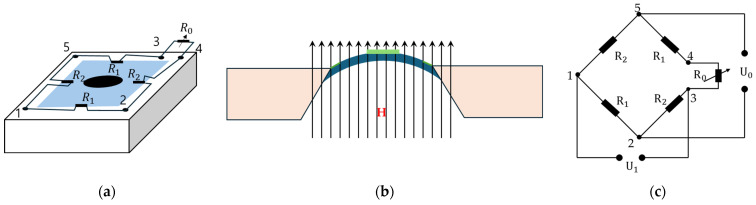
Schematic configuration and working principle of the magnetometer based on a MRE; (**a**) schematic diagram of magnetic sensor, (**b**) the sensor’s cross section indicating the direction of the magnetic field H, (**c**) circuit diagram of the Wheatstone bridge [76].

**Figure 12:**
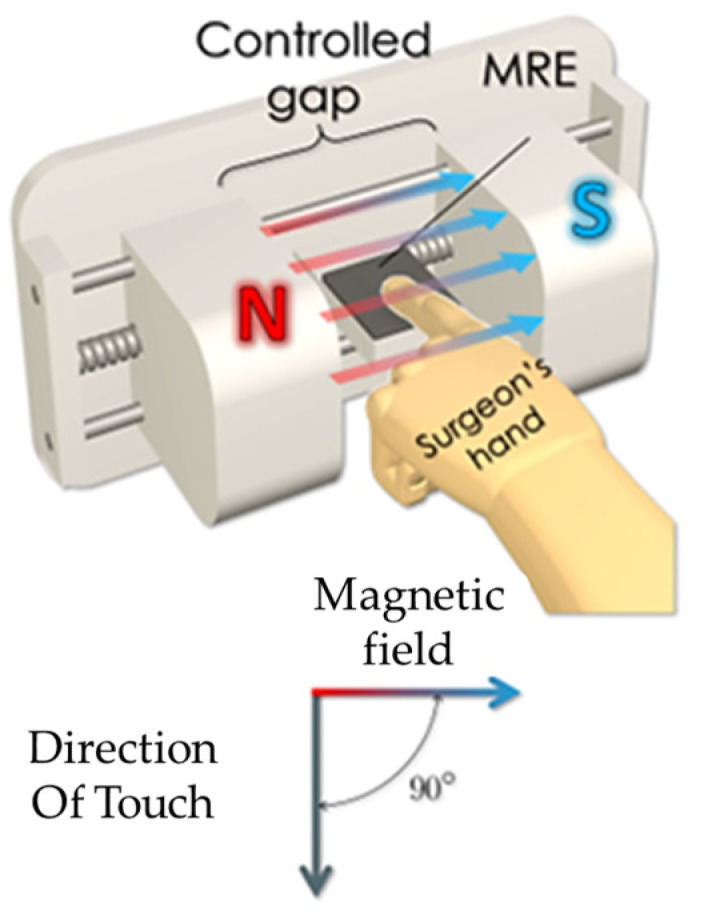
Schematic configuration the MRE force sensor [81]. Reproduced with permission from [Amir Hooshiar], [Materials Science and Engineering C]; published by [Elsevier], (2009).

**Figure 13:**
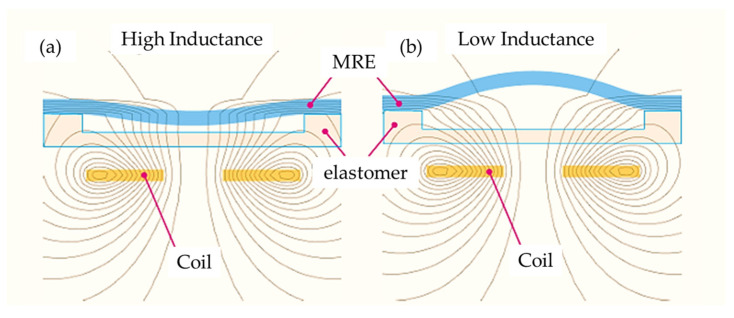
Working principle of the inductive sensor with flat coils [82]. (**a**) high inductance with a small deformation of the MRE skin, (**b**) low inductance with a large deformation of the MRE skin. Reproduced with permission from [Hongbo Wang], [International Conference on Soft Robotics (RoboSoft)]; published by [IEEE], (2009).

As is well known, conventional magnetorheological elastomer (MRE) is composed of micro-sized magnetizable particles, a matrix and additives considering high saturation magnetization, high permeability and low remnant magnetization of the particles such as CIP. Since the resistance of the ERP is controllable by the magnitude of the magnetic intensity, it can be applicable to several types of sensors, such as a pressure sensor. Kang et al. [71] proposed a measurement method for the field-dependent pressure drop with and without the cavitation in MR dampers. It has been identified that the low initial pressure causes cavitation, resulting in an abnormal damping force versus displacement and damping force versus piston velocity. In addition, it has been visually observed that the phase delay of the damping force is increased as the stroke increases during extension since the internal pressure of the damper is too low. Song et al. [72] developed a haptic interface to provide a passive feedback force with high force fidelity and low inertia. As for the slave robot, a catheter and a guidewire can be navigated simultaneously, which allows the two degrees of action. The resistance force of the catheter navigation is then measured and reflected to the user through the master haptic interface. It has been experimentally demonstrated that the proposed feedback device provides not only the haptic feedback, but also captures the surgeon’s manipulation of the catheter by applying the magnetic field to the detection circuit. Bhat et al. [73] wrote a review article of MRF-based reliability devices from the perspective of modeling, sensors and control strategies. In particular, several types of rehabilitation devices including knee sleeves, prophylactic braces, functional braces, knee and angle orthoses with MRF dampers were reviewed. In addition, several types of sensors associated with MRF-based rehabilitation devices were introduced and their sensing characteristics were discussed, including a flexible resistive sensor, body-oriented sensor, multi-measurement sensor and mechanical sensor. Li et al. [74] proposed a new force sensor using a MRE which has three components: a mechanical unit, electrical circuit and LED display unit. Figure 10 presents the schematic configuration and working principle of the MRE force sensor. It is seen that mechanical design consists of eight components. The base holder is used to connect all parts together and separate the electrodes, while the lower plate is fixed on the base holder with a strong adhesive. The MRE sample is fixed between the lower plate and the button. When the upper plate is screwed, the rubber O-ring can provide the button with a preload that is adjustable by screwing or unscrewing. Therefore, the preload can be applied on the MRE sample for a primary deformation and the metal spring is pressed to obtain a primary deformation, thus yielding a spring that provides a recovery force for the sensor. This sensing concept has been proved by formulating the relationship between the voltage and external loading (force). Faidley et al. [75] investigated the possibility of using a MRE as a sensing mechanism by making a 5 mm thick MRE sample consisting of silicone rubber and CIPs 9 mcm in diameter. As for the testing samples, both the initial bulk magnetization and the length-aligned MREs are used to distinguish the different behavior. It is noted that the magnetization of each particle is driven closer to the direction of the applied field so that they become more closely aligned with each other which results in the decrement of the dipole–dipole interaction between the neighboring particles. Thus, as the sample is stretched and its cross-sectional area inside the pick-up coil decreases, the magnetic flux in the coil changes. Now, this change in flux induces a voltage in the pick-up coil. In other words, a strong correlation between the output-induced voltage and the input strain rate makes the materials very promising for large strain, non-contact strain-rate sensors and force sensors in the proportional alignment region. Du and Chen [76] developed a low-cost magnetometer based on the magneto-strictive effect of MREs. The micromechanical sensor consists of a silicon sensitivity diaphragm embedded with a piezoresistive Wheatstone bridge and a MRE layer attached to the diaphragm. Figure 11 presents the schematic configuration and working principle of the magnetometer based on a MRE layer. It is seen from Figure 11a that the magnetic field sensor consists of a silicon cup structure and disk-like MRE layer attached to the sensitivity diaphragm (SD). The silicon cup has four piezoresistors placed along the periphery of the SD and connected with a Wheatstone bridge configuration. The sensors’ cross-section with the magnetic field is shown in Figure 11b, where the SD will move to a displacement due to the magnetic field. Figure 11c shows a simple circuit diagram of the Wheatstone bridge, where R2 and R1 are the piezo-resistors perpendicular and parallel to the SD edge. It is noted that Figure 11a,c denote the node marks. It has been found from this work that the proposed sensor has a good linearity in the magnetic field range of 0–120 kA/m, but the saturation occurs above 150 kA/m. In addition, it was also observed that the remaining magnetostriction of the MRE decreases and the repeatability becomes better. Some future works on the optimal shape, optimal concentration of the magnetic particles and optimal size are to be further explored to demonstrate if they can be applicable as practical sensing devices. Ghafoorianfar and Gordaninejad [77] proposed a wireless MRE sensor which is capable of sensing compression and shear. The MRE sensor system consists of a disk-shape MRE sample with two thin steel electrodes attached to both sides and two wires connected to electrodes to obtain the change in the electrical resistance from the piezo-resistance behavior of the MRE when various axial and shear stresses are applied. It has been found from experimental results that two orders of the magnitude change in resistance under an 8% compressive strain is achieved and the MRE sample with 35 vol.% of the iron particles exhibits more than 30 times increase in resistance, applying up to 100% shear strain. Behrooz et al. [78] presented an adaptive bridge bearing which can sense structural loads and tune its properties to mitigate unwanted vibrations using MRE layers in which the stiffness, structural wind and traffic load are dependent on the magnetic field. After fabricating a protype of a MRE sample combined with shear, compression and electrical resistance, MRE compression tests were undertaken. It has been shown that the force-displacement loops increase as the magnetic field increases and the strain of the MRE increases from 10% to 50% and the stiffness increases from 24.3 N/m to 27.6 N/m with the increasing frequency. Therefore, a robust bearing design can be achieved by controlling the magnetic field dependent on the stiffness of the MRE layers that ensures the desired performances. Qia et al. [79] designed a self-powered magnetic field sensor using a MRE and triboelectric nanogenerator (TENG) which can be used for both time-varying and uniform magnetic field sensing. This sensor relies on the contact electrification and electrostatic induction of TENG to generate an electrical signal responding to the magnetic induced deformation of MRE without any external power or stimuli. It has been shown that the proposed sensor exhibits a fast response time of 20 ms and maximum sensitivity of 16 mV/mT with 60 wt.% MRE. It is noted that the sensitivity and detecting range can be adjusted by changing several parameters of the sensing device. Li et al. [80] fabricated a new self-sensing bearing based on an anisotropic MRE consisting of a multi-walled carbon nanotube, CIPs with polydimethylsiloxane matrix and the self-sensing characteristics of the MRE such as multi-field coupling of the load and magnetic fields. Then, it was applied to a vibration system to reduce vibrations up to 30%. Therefore, the results presented in this work indicate that the modified MRE bearing could be simultaneously used as an actuator and a sensor. Hooshiar et al. [81] proposed a stiffness display using a MRE which can be applicable to manual surgery or robotic minimally invasive surgery. Figure 12 shows a schematic configuration and working principle of the proposed display device. In this work, a rubber-based bi-layer composite MRE was used to alleviate the nonlinear contact problem of the soft material and a pair of permanent magnets were employed to generate the magnetic field. A slim sheet of the composite MRE was placed at the mid-span of two permanent magnets and two DC servo motors were used for controlling the magnetic field by changing the gap distance between the magnets. In addition, a force sensor was placed to measure the touch force in a proportional–integral–derivative (PID) controller implementation in a closed-loop manner. It has been shown from experimental tests that the range of the tactile forces and stiffness are within the favorable range from 8% to 16%, as reported in previous works. Wang et al. [82] proposed a wireless inductive sensing device using a MRE to measure the body deformation of soft pneumatic actuators (SPAs). Figure 13 presents a working principle of the inductive sensor for SPAs with flat coils. As seen from the figure, the stretchable MRE skin of the soft actuator plays a role in the nonconductive ferromagnetic material which can be magnetized and demagnetized in a very low magnetic field which can increase the total inductance of the coil-MRE system. When the MRE skin is deformed, the average distance between the MRE skin and coil changes and hence the inductance is varied. Therefore, the deformation of the MRE skin can be monitored wirelessly by measuring the inductance of the coil. It has been demonstrated that the deformation is proportional to the pressure and the inductance of the bending angle of the SPA sample is decreased during an inflation and deflation cycle.

**Figure 14:**
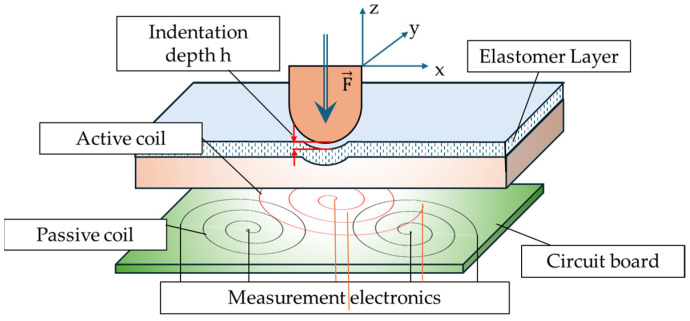
Schematic design and operating principle of the MRE force sensor [84].

**Figure 15:**
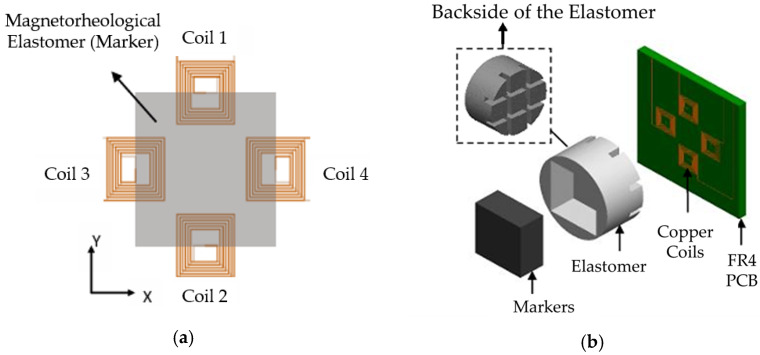
Schematic design of a three-axes inductive tactile sensor; (**a**) top view of MRE sensor (**b**) exploded view of MRE sensor [85]. Reproduced with permission from [Muhammad A. Khalid], [IEEE Sensors Journal]; published by [IEEE], (2022).

Shabdin et al. [83] proposed a graphite (Gr)-based MRE (Gr-MRE) to evaluate the effect of Gr to the resistivity under applied forces and magnetic fields. It has been found from this evaluation that two relationship curve resistances (*R*) under different applied forces (*F*) and different magnetic fields (*B*) are obtained. Then, it has been identified from the relationship curves that the presence of a Gr fraction arrangement contributes to the conductivity of the Gr-MRE and the field-dependent modulus can be improved, particularly at low strain amplitudes, compared with conventional MRE without the addition of Gr. In addition, from the relationships between force and field-dependent resistivity, the Gr-MRE can be one of potential force sensors. Gast and Zimmermann [84] investigated the capability of a tactile sensor using a MRE, especially to determine the position of indentation by measuring the inductance of multiple planar coils and a soft magneto-sensitive layer. Figure 14 shows the working principle of the proposed tactile sensor with the magnetic circuit coil board. In the figure, the indentation of depth *h* is the negative z direction and hence the indentation is in a certain interval of the *x* and *y* position. The inductance of the magnetic coil is increased by increasing the magnetic flux intensity, and the variables of *h*, *x* and *y* are changed or controlled. Therefore, the inductance change curve of a planar-shifted indentation with a constant depth of *h* can be determined by identifying the peak value (or resonance) of the induction change from the measurement electronics, which is the inductance-to-digital converter operating in a single channel mode which ensures an adjustment of the frequency to maintain the resonance in the sense of the inductance change. Khalid et al. [85] proposed a three-axis inductive tactile sensor using a MRE which is applicable to surgical robotic systems. Figure 15 presents the schematic configuration of the sensor and working principle. The sensor consists of copper coils on a printed circuit board and patterned elastomer layer between the marker and coils where the marker is the mixture of a material with high magnetic permeability and the elastomer. The markers and coils are separated by a layer of the elastomer such that there is an initial gap between the coils and markers. When a force is applied on the sensor’s surface, the marker displaces from the initial position, changing either the overlap area between the marker and the coils or the distance between them. This change in the position of the marker causes a change in the resonance frequency of the LC circuit through which the inductance is calculated. Therefore, the applied force can be estimated by measuring the change in the inductance values. It is noted that the direction of the applied force can be also identified by configuring the positions of the coils. Tasin et al. [86] made a new type of MRE to enhance the storage modulus, magnetostriction magnitude and reaction (normal) force to investigate a potential application of the proposed MREs as sensors or sensing devices. The proposed MRE samples consist of a polydimethylsiloxane (PDMS) silicone rubber and high CIPs which have an average diameter of 5 mcm with a spherical shape to have specific characterizations including high saturation magnetization, high permeability, high normal force and low remanent magnetization. It has been shown that the target specifications are achieved from experimental measurements and it is noted that force sensors and pressure sensors could be devised based on the results presented in this work, since moderate stiffness (or midrange modulus) could ensure the high durability of the sensors while maintaining sufficient magnetostriction magnitude. Costi et al. [87] presented the first case of a 3D-printable self-sensing MRE and tested cyclic mechanical compression and tensile mode analysis at a high deformation rate up to 20%. The MRE samples containing the styrene-based thermoplastic elastomer (TPS) were fabricated at 0.5–2.0 Tesla. In addition, carbon black (CB) fillers were added to have two properties of the MR effect and piezoresistive effect, providing a self-sensing capability. It has been shown that several properties including the magneto-resistive, magneto-strictive and hall effects could be achievable by selecting optimal ratios between the CIP concentrations and amount of the fillers as a function of the magnetic field. Therefore, various types of self-sensing devices which have diverse shapes can be simply made from the 3D printing technology.

**Figure 16:**
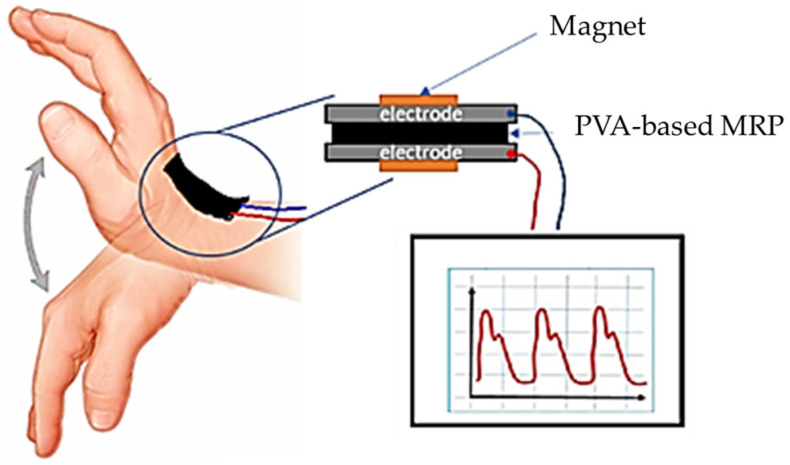
Schematic design of the MRP force sensor [92]. Reproduced with permission from [Norhiwani Mohd Hapipi], [Sensors]; published by [MDPI], (2021).

Recently, a magneto-sensitive viscoelastic polymer consisting of low cross-link polyurethane (PU) and magnetic particles, called a magnetorheological plastomer (MRP), has been actively studied in the sense of the development of an enhanced MRP and its various applications, especially sensor applications. Xu et al. [88] experimentally investigated the flow motions operated under the squeeze flow mode of a MRP, including the elastic deformation region, stress relaxation region and plastic flow region. It was found from this work that the field-dependent compressive and tensile yield stress are sensitive to the magnetic field, which provides a possibility of the sensor fabrication of the proposed MRP consisting of a CIP and plastic polyurethane matrix. Xuan et al. [89] surveyed the state-of-art on the science and technology levels of the MRPs. Several different types of MRPs were introduced and their field-dependent characteristics were discussed in the sense of their application as sensors. The types of MRPs are classified by the mixing materials (matrix): silica gel, polymer gel, hydrogel, cross-linked polyurethane, triblock copolymer polystyrene and petroleum jelly. In addition, several properties of MRPs such as deformable shape, field-dependent conductivity and MR effect depending on the magnetic field were discussed for various applications as actuators or sensors. Xu et al. [90] proposed a new MRP whose resistance can be controllable and hence can be used as a strain sensor. As a first step, a carbon filler doped MRP samples with carbon micro-fibers, carbon nanotubes and their mixtures were made to observe both the ME effect and magnetic field-dependent electrical property. It has been shown that the vibration range of the resistance is increased by increasing the oscillation amplitude and the period of the resistance is half of the period of the strain, showing possible application to multifunctional properties including sensors. Qi et al. [91] developed a versatile MRP based on polycaprolactone (PCL) and thermoplastic polyurethane (TPU) which can have 3D printability, switchable mechanics, shape memory and self-healing properties. After characterization of the proposed MRP, several features, which are achievable features of the proposed MRP such as static and dynamic viscoelasticity, shape memory property and self-healing properties, were presented and discussed. Hapipi et al. [92] proposed a new type of MRP based on polyvinyl alcohol (PVA-based MRP) which possesses an excellent MR effect which is a potential candidate as an actuator. In addition, the proposed MRP produces a shear stiffening (ST) effect which is very beneficial in fabrication pressure strain sensors. The PVA-based MRP exhibits an ST behavior, which is a common phenomenon in which the viscosity increases when the materials are exposed to external stress beyond their critical shear rate. Both excellent flexibility and conductivity are good properties to fabricate pressure strain sensors. Figure 16 shows the schematic of the pressure or strain sensor to detect human wrist motions like bending and stretching utilizing the PVA-based MRP. Since MRP is flexible, it can be applicable to several soft systems where the pressure strain needs to be sensed as an output or a feedback signal in the closed loop control systems. Xu et al. [93] proposed a flexible self-powered magnetism/pressure dual-mode sensor consisting of a MR plastomer (MRP) in which the proposed pressure sensor is sensitive to a slight pressure (1.3 kPa) as well as responsive to a small magnetic field (12 mT). The working mechanism of this sensor is based on the displacement reaction of Fe and CuSO_4_, and the micro-scale carbonyl iron (CIP) in the MRP electrode aggregates into the chain-like structures resulting in the enhancement of the electrochemical activity of ions in the electrolyte of the electrode materials. Zaini [94] used a graphite to fabricate a MRP which has a sensing capability owing to its flexibility, soft nature, responsiveness to external magnetic field and conductive electricity. The main goal of this work was to investigate the electrical properties of MRPs such as the conductivity absolutely required to use them as potential sensors. It has been found from experiments that the conductivity of 10 wt.% graphite is increased up to 178.06%, which is the highest at a magnetic flux density of 400 mT compared to without the graphite. The wide change in the conductivity and the sensitivity of the proposed MRP depending upon the magnetic field intensity can contribute to the potential applications of the sensing detection devices. Hapipi et al. [95] fabricated the polyvinyl alcohol (PVA)-based MRP to enhance the MR effect as well as shear stiffening (ST) effect by the magnetic field. After the fabrication of several samples by varying the solvent ratios of the binary solvent mixture dimethyl sulfoxide (DMSO) to water, the field-dependent properties were tested. It has been identified that the MR and ST effects are dependent on the solvent ratios and hence appropriate actuators and sensors could be devised by adjusting both the field intensity and solvent ratios. Amlee et al. [96] proposed a hydrogel-based MRP (HMRP) with a certain amount of the graphite to use as a force sensor. In this work, HMRPs with 0 wt.% up to 15 wt.% were prepared and tested under applied forces ranging from 0 N to 5 N. It has been shown that the force can be measured from the resistance change of the HMRP with and without the magnetic field for practical feasibility in the medical area for physiology or therapy. Pang et al. [97] fabricated a new type of MRP using a series of hollow powders (HGP) to enhance the impact resistance of the material and dynamic compressive properties under a high strain rate. It was shown that the magnet-induced yield stress increased from 7.3 MPa to 17.1 MPa by adding 9 vol.% CIP. However, it was also found that this benefit could not be achieved when the mass ratio of the HGP was larger than 0.67. This directly indicates that the volume fraction of the CIP and HGP is equally significant to obtain the advanced properties of the proposed MRP so that it can be applicable to sensors and actuators.

## 5. Summary and Conclusions

In this review article, sensors or sensing devices using electrorheological fluids (ERFs) and magnetorheological materials (MRMs) which are classified into magnetorheological fluids (MRF), magnetorheological elastomers (MRE) and magnetorheological plastomers (MRP) have been surveyed and their measurement characteristics have been discussed in the sense of their working principles and measurements, as well as physical parameters via the calibration between sensor signals and physical parameters. One of most popular sensors using the proposed smart materials is a force sensor which can be easily fabricated from a relationship between the input field (electrical voltage or magnetic field) and a simple electrical circuit representing the output resistive signals which can produce the force from the field-dependent stiffness and damping. Moreover, strain sensors, displacement sensors and magnetic measurement devices were also proposed by several researchers. In fact, there are several excellent properties of the proposed materials to make several different types of sensors which are physical parameters including stress, modulus, viscosity, stiffness and damping, and these are controlled as a function of the input field. This unique property can produce a great potential to devise many different sensors, including accelerometers.

One of the critical requirements for an effective sensor or sensing device is the response time (bandwidth) of the sensing signal by the input source. It has been reported in several research works that the signal response times of ERF/MRF sensors or sensing devices are fast, while those from MRE/MRP sensors or sensing devices are relatively slow. This is because the field-dependent particles of ERFs/MRFs are moved in carrier oils without any constraint, but the dynamic motion of the field-dependent particles embedded in MREs/MRPs is limited due to the solid structures. More specifically, it is known that the response time of ERF and MRF sensors is around 1 ms and 5 ms, respectively. The reason why the response time of ERF sensors is faster than MRFs is the difference in the particles. The mass density of the particles (in a general polymer) of ERFs is much lower than that of the particles (in general iron) of MRFs and hence move faster in carrier oil zones. On the other hand, it is known that the response time of MRE/MRP sensors is around 50 ms. It is noted here that the response times mentioned above mostly depend on the magnetic field intensity. Therefore, the response times can be faster or slower with different particle materials, different particle sizes, different particle shapes, different chain formations of the isotropic and anisotropic and different additives. It should be remarked that there is a trade-off behavior between the response time and sensed signal intensity. Therefore, several researchers are currently working on an optimization methodology to achieve both a fast response time and high sensitivity of sensors or sensing devices made from smart materials associated with ERFs, MRFs, MREs and MRPs. Due to the difference in the response time and sensitivity, each sensor has advantages and disadvantages. ERF-based sensors can measure the high frequency signal in dynamic applications and very sensitive signals in tactile array applications, but these sensors have a couple of limitations. For example, is the sensor is applicable to low force measurement only with a high voltage input. MRF-based sensors are applicable as ERF-based sensors with a little lower sampling signal frequency. However, they can be used to measure high forces generated in application systems such as the haptic master and in the identification of unknown models of MR dampers. On the other hand, MRE/MRF-based sensors can be applicable to relatively low frequency systems due to their slow response time. However, they have a couple of advantages compared with ERF/MRF-based sensors: they can be applicable to measure the signals (for example, force) of flexible systems, and they have better durability since there are no fluid leakage problems during long-term operations.

Despite numerous works on sensors or sensing devices, so far there is no commercially available sensors or sensing devices made from these smart materials. There are several issues to be resolved for this target. The most significant problem is the lack of a standard calibration procedure. For example, the electric signals from the sensors need to be converted to physical parameters using proper calibration factors which should be provided by sensor manufacturing companies. Currently, most of the sensors introduced in this review articles do not have a standard or general calibration factor. Moreover, in several works, in order to convert from electric signals to the physical parameters, several calibration factors are suggested to match with the physical parameters measured from commercial sensors. Therefore, in order to bring smart material-based sensors to a real marketplace, more studies on the rating the sensors’ specifications with generalized calibration factors like strain gauges should be carried out as soon as possible. In addition, detailed specifications to satisfy sensors’ requirements including high accuracy, high precision, small resolution, long repeatability, high sensitivity, easy calibration, temperature compensation and optimized electrical circuits need to be explored in depth. An optimal fabrication of the material which possesses a high adaptability to sensing devices instead of an actuating mechanism is also crucial to achieve a high performance for the sensors and sensing devices. It is suggested that one way to accomplish such specifications is to have advanced materials adaptable to sensors be integrated to a microcircuit using modern technologies. For example, a neural network integrating artificial intelligence and a fuzzy algorithm can be used to determine the principal ingredients of the smart materials such as volume of the particles, viscosity of the carrier liquids, proper additives and sensitive electrical circuits. It is also noted that big data analysis may be utilized to achieve several relationships among important parameters for sensing devices: the input fields, output sensing signals, signal convertors and calibration factors.

## Data Availability

The raw/processed data required to reproduce these findings cannot be shared at this time as the data also form part of an ongoing study. In future, however, the raw data required to reproduce these findings will be available from the corresponding authors.
